# Progress towards dog-mediated rabies elimination in PR China: a scoping review

**DOI:** 10.1186/s40249-023-01082-3

**Published:** 2023-04-06

**Authors:** Tianren Shen, Susan Christina Welburn, Long Sun, Guo-Jing Yang

**Affiliations:** 1 Key Laboratory of Tropical Translational Medicine of Ministry of Education, The School of Tropical Medicine, The First Affiliated Hospital, Hainan Medical University, Haikou, 571199 Hainan People’s Republic of China; 2 Zhejiang University-University of Edinburgh Joint Institute, Zhejiang University, International Campus, 718 East Haizhou Road, Haining, 314400 People’s Republic of China; 3 Infection Medicine, Deanery of Biomedical Sciences, Edinburgh Medical School, College of Medicine and Veterinary Medicine, The University of Edinburgh, 1 George Square, Edinburgh, EH8 9JZ Scotland, UK; 4 Department of Infectious Diseases, The First Affiliated Hospital of Hainan Medical University, Hainan Medical University, Haikou, Hainan People’s Republic of China

**Keywords:** Rabies, Lyssavirus, China, Elimination, Dog, Epidemics, One Health, Neglected, Tropical, Underreporting

## Abstract

**Background:**

Rabies continues to be a serious threat to global public health endangering people’s health and public health safety. In the People’s Republic of China, multi-sectoral and comprehensive prevention and control strategies have aimed to extensively curb human rabies transmission. Here, we examine the current state of rabies infection in China, explore strategic interventions put in place in response to WHO’s ambition of “Zero rabies deaths by 2030” and critically assess the constraints and feasibility of dog-mediated rabies elimination in China.

**Methods:**

This study analyzed and evaluated the process towards dog-mediated rabies elimination in China from five perspectives: namely, human, dog, policy, challenge, and prospects. Evidence-based data on progress of dog-mediated rabies elimination in China was derived from a number of sources; a literature search was undertaken using PubMed, Web of Science and CNKI databases, distribution data for human rabies cases as derived from the Data-center of the China Public Health Science and policy and document data were obtained from official websites of the relevant China ministries and commissions.

**Results:**

The incidence of human rabies cases in China have shown a downward trend year-on-year since 2007. Implementation of a government-led, multi-sectoral “One Health” approach to combating rabies has driven down the total number of rabies deaths nationwide to around 200 in 2020. The number of provincial-level administrative divisions (PLADs) reporting human cases of rabies has also decreased to 21 in 2020, 13 of which reported less than 10 cases. Furthermore, the number of outpatient visits seeking rabies post-exposure prophylaxis has risen dramatically over the past two decades, with demand being 15 times higher than it was initially. There remain however, significant gaps in rabies elimination outcomes across the different regions of China. To date the target of achieving a canine rabies vaccination rate of > 75% has not been met. The challenges of rabies immunization of dogs and dog management in underdeveloped cities and rural areas need to be addressed together with more effective animal surveillance and rabies risk from and too wildlife and livestock.

**Conclusions:**

The Chinese government-led, multi-sectoral “One Health” approach to combating rabies and has made significant progress over the past decade. Development and adoption of more cost-effective One Health strategies can achieve more nationally beneficial rabies elimination outcomes. The ambitious target of “Zero rabies deaths by 2030” can be met through establishment of long-lasting herd immunity in dogs by means of dog mass vaccination campaigns, dog population management, epidemiological surveillance and the application of large-scale oral rabies vaccine to eliminate rabies in wild animals coupled with deployment of cost-effective human post-exposure prophylaxis, and community education.

**Graphical Abstract:**

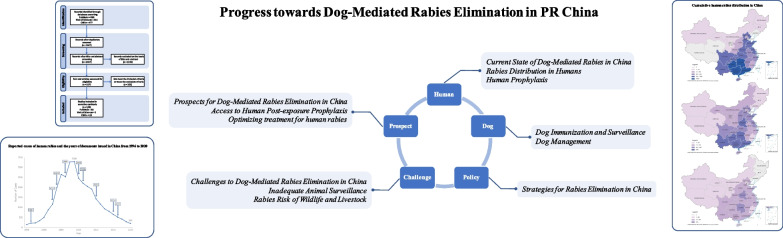

## Background

Rabies is a lethal zoonotic encephalomyelitis caused by infection with the rabies lyssavirus, a negative-strand RNA virus in Rhabdoviridae family in the order Mononegavirales [1, 2]. Rabies is one of the oldest zoonotic diseases of humankind [3] with the earliest record of infection appearing in a code of laws in the state of Eshnunna (Mesopotamia) in approximately 1930 B.C. that instructed owners of dogs showing symptoms of rabies to do what was necessary to prevent the dog from biting the owner or others [4]. The earliest report of rabies in China can be found in an ancient text called Zuozhuan; *‘In (556 BC), people in the state of Lu drove away mad dogs’*.

Rabies, as a neglected tropical disease (NTD), is a serious global public health problem with 99% of rabies deaths occurring in low- and middle- income countries in which dog-mediated rabies is endemic. The disease is responsible for an approximate 59,000 human deaths, 3.7 million disability-adjusted life years (DALYs) and 8.6 billion economic losses worldwide every year, with 95% of cases reported in Asia and Africa [5]. Rabies disproportionately affects children, with 40% of victims being children under 15 years old [6] and once clinical signs of rabies manifest a rabies infection is always lethal. As for most of the NTDs, rabies is a disease of poverty and rabies infections and rabies deaths are largely underreported within health systems; the underreporting rate of rabies, infection vs death is estimated at 20 fold in Asia and 160 fold in Africa [7]. Early infection can be misdiagnosed as another viral encephalitis or as cerebral malaria (where malaria is endemic) [8] and more than 75% of rabies deaths occur at home, undiagnosed and not reported to the health system [9].

Rabies has long been prevalent across China. In 1934, it was reported that an estimated 5000 people were dying from rabies, annually in China [10] although prior to 1950, prevalence and incidence data were incomplete. In the 1950s, the Chinese government established the National Notifiable Infectious Disease Reporting Information System (NIDRIS) listing rabies as a class B infectious disease with mandatory reporting. Since inception of NIDRIS, the People’s Republic of China (PR China) has reported 132,568 cases of human rabies [11] and three major resurgences of rabies [12] with peaks of infection in the 1950s, 1980s, and early 2000s. The first resurgence of rabies in PR China occurred between 1950 and 1959, reaching a peak of infection in 1956, with 1042 cases reported. At this time the population size was low and there were a relatively small number of dogs per capita, conditions were poor and by 1959 famine was reported to be affecting one third of all provinces. An imperfect NIDRIS may also have contributed to underreporting of cases.

The second period of resurgence for rabies in PR China occurred from 1960 to 1995. Between 1979 and 1989 the annual number of cases remained above 4000 for 11 consecutive years, reached a historical peak of 7037 cases in 1981, and subsequently decreased year on year into the 1990s. During this period rabies was widely distributed across China, with a high incidence in the southern provinces. From 1976, with rapid economic growth, the dog population increased significantly, causing a rise in the number of rabies cases reported [13].

The third resurgence of rabies cases began in 1996 and continues to this day. From 2000, the number of rabies cases increased rapidly, peaking at 3300 cases in 2007 at and decreasing year on year thereafter. The increasing number of dogs kept by urban residents and in rural communities, rapid urbanization in China and a highly developed transportation network associated with rapid economic development in China are considered to have contributed to the spread of rabies [14].

Rabies remains a significant public health threat in China with around 40 million people injured by dogs every year [15] with 99% of human rabies cases being transmitted by dogs [16]. In 2020, the number of rabies deaths ranked 4th amongst 39 reportable infectious diseases ranking just below HIV/AIDS (Human Immunodeficiency Virus/Acquired Immune Deficiency Syndrome), tuberculosis, and viral hepatitis.

Despite rabies exhibiting a broad global spatial distribution and being present in many animal species, it is considered feasible to eliminate dog rabies and dog-mediated human rabies using existing and effective vaccines in humans and dogs. The World Health Organization (WHO), the World Organization for Animal Health (WOAH); the Food and Agriculture Organization of the United Nations (FAO) and the Global Alliance for Rabies Control (GARC), launched a global framework for rabies in December 2015 and a global strategic plan in 2018 towards eliminating dog-mediated rabies by 2030 [17–19].

Human and dog rabies are closely correlated, and rabies elimination is well suited to the application of a One Health approach. One Health is an integrated unifying approach that recognizes that the health of humans, domestic and wild animals and our ecosystem are closely linked and interdependent. A One Health approach for rabies would include implementing mass dog vaccination, improving post-exposure prophylaxis accessibility on humans, management of infection at the human, domestic animal and wildlife interface and building awareness of rabies in local communities.

The Chinese government has established a government-led, multi-sectoral “One Health” approach to combating rabies and has made significant progress over the past decade. In 2012, Chinese Center for Disease Control and Prevention (China CDC), proposed a road map for the elimination of rabies in China [20]. The elimination plan for dog-mediated rabies in China proposed three progressive phases. The first or preparation phase incorporated in the Twelfth Five-Year Plan was implemented between 2011 and 2015 and aimed to decrease the number of reported cases in 2015 by more than 50% compared with 2010 (setting a target of around 1000 rabies cases). The workplan included; establishment of multi-sectoral coordination, refining legal, technical and veterinary systems and securing animal vaccines.

The second phase, a Five-Year Plan running from 2016 to 2020 aimed to decrease the number of reported cases in 2020 by more than 90% compared with 2010, setting a target of around 200 rabies cases) and focusing on strengthening the rabies surveillance-response system.

China achieved the first two phases with the number of human reported cases maintained at around 200/annum but fell short of the target of achieving a canine rabies vaccination rate of > 75%.

The third and final elimination phase will operate between 2021 and 2025, and aims to decrease the number of dog-mediated rabies cases to zero. During the Fourteenth Five-year Plan, China CDC will implement a series of comprehensive prevention and control measures, undertake rabies elimination certification and apply large-scale oral rabies vaccine to eliminate rabies in wild animals.

In this scoping review, we reviewed published literature and available information on rabies from China, to establish an evidence base with regards to the current state of the prevalence and distribution of rabies the progress of human exposure prophylaxis, the level of herd immunity, the surveillance on dogs and other animals, the strategies for rabies elimination in China. Our primary objective was to summarize current data to aid in the development of a national rabies action plan aimed at achieving the target of zero human death from dog-mediated rabies by 2030.

## Methods

### Information sources and search strategy

This review follows the guidelines of the PRISMA-ScR (Preferred Reporting Items for Systematic Reviews and Meta-Analyses Extension for Scoping Reviews) statement for scoping reviews [21]. A wide range of resources were used to obtain evidence-based information on dog-mediated rabies elimination in China. For the literature search, PubMed, Web of Science and CNKI (in Chinese) (China National Knowledge Infrastructure, https://www.cnki.net/) were searched from inception to November 20, 2021, for relevant articles. For PubMed and Web of Science, all fields/topics keywords: (“Rabies” OR “Rabid”) AND “China” were searched. For CNKI, the main subject term: “Rabies” with a “core journal” filter was searched.

### Inclusion and exclusion criteria

Published papers were eligible for inclusion if they focused on relevant topics: (1) China rabies epidemic; (2) transmission and surveillance of rabies in animals (and wildlife) of China; (3) China rabies policy; (4) burden of rabies in China.

Exclusion criteria were defined as follows: (1) cases of misdiagnosis of rabies; (2) cases of rabies infection in single individuals; (3) studies on transmission of rabies due to organ transplantation; (4) epidemiological statistics for short time periods in single province or city; (5) study design was biased; (6) inaccessible for full text; (7) duplicated studies.

### Study screening, data extraction and analysis

**Fig. 1:**
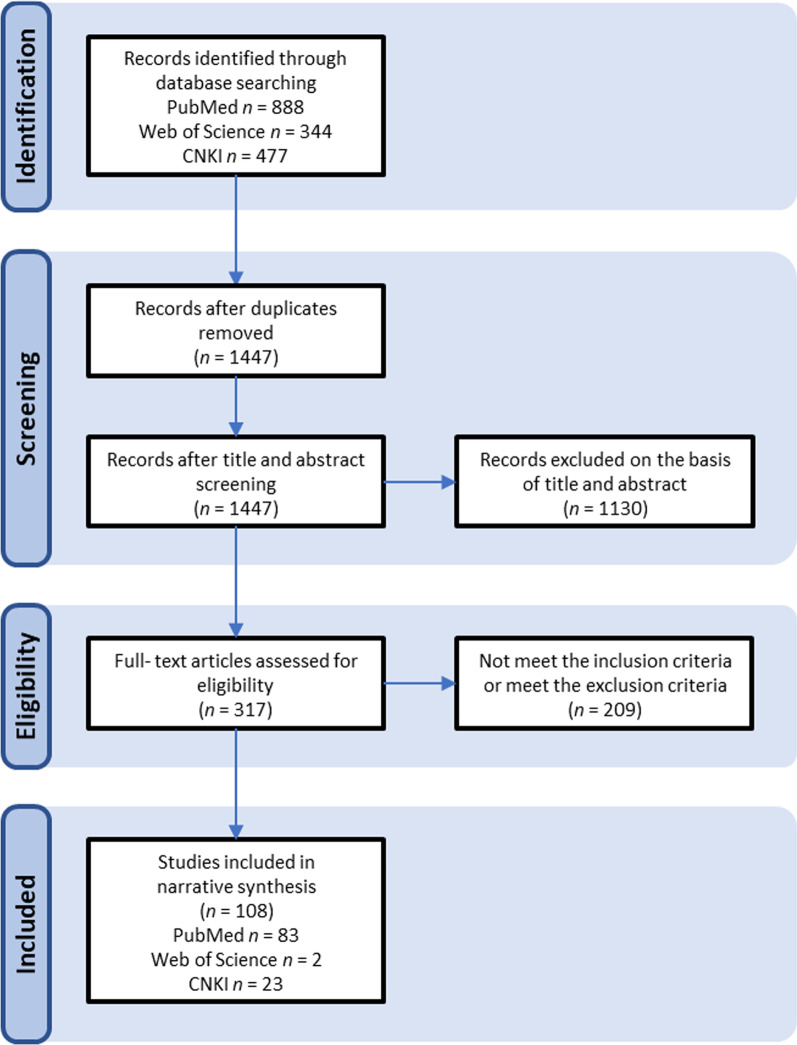
Flow diagram of literature screening and selection process

References from the initially identified studies were also reviewed and included in this review if relevant. For each eligible document, a full-text review and a narrative synthesis was made, which was then further digested into a qualitative review. Data on the distribution of human rabies cases were obtained at The Data-center of China Public Health Science. Policy and document data are obtained from the official websites of China ministries and commissions. The results of the screening and selection process are presented in Fig. 1.

## Results

### Human rabies in China

#### Current state of dog-mediated rabies in China

Following the Severe Acute Respiratory Syndrome (SARS) outbreak in 2003, China established a national direct network reporting system for infectious disease epidemics and public health emergencies in 2004 and was put into use nationwide [14] shortening the average information reporting time from 5 days to 4 h. In August of 2004, the Law on the Prevention and Control of Infectious Diseases was passed, establishing a legal framework for the publication of information on the epidemic situation of infectious diseases.

**Fig. 2:**
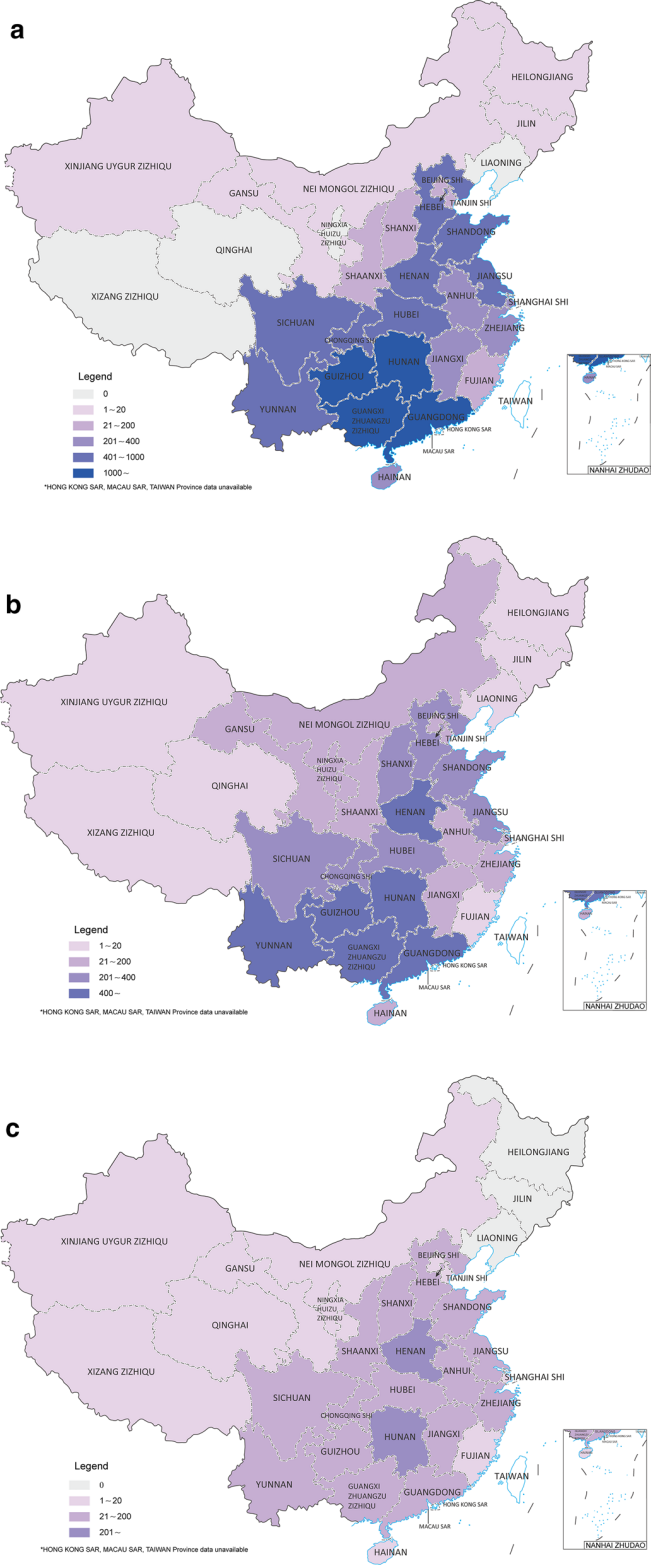
Cumulative human rabies distribution in China. **a** 2006–2010; **b** 2011–2015; **c** 2016–2020

**Fig. 3:**
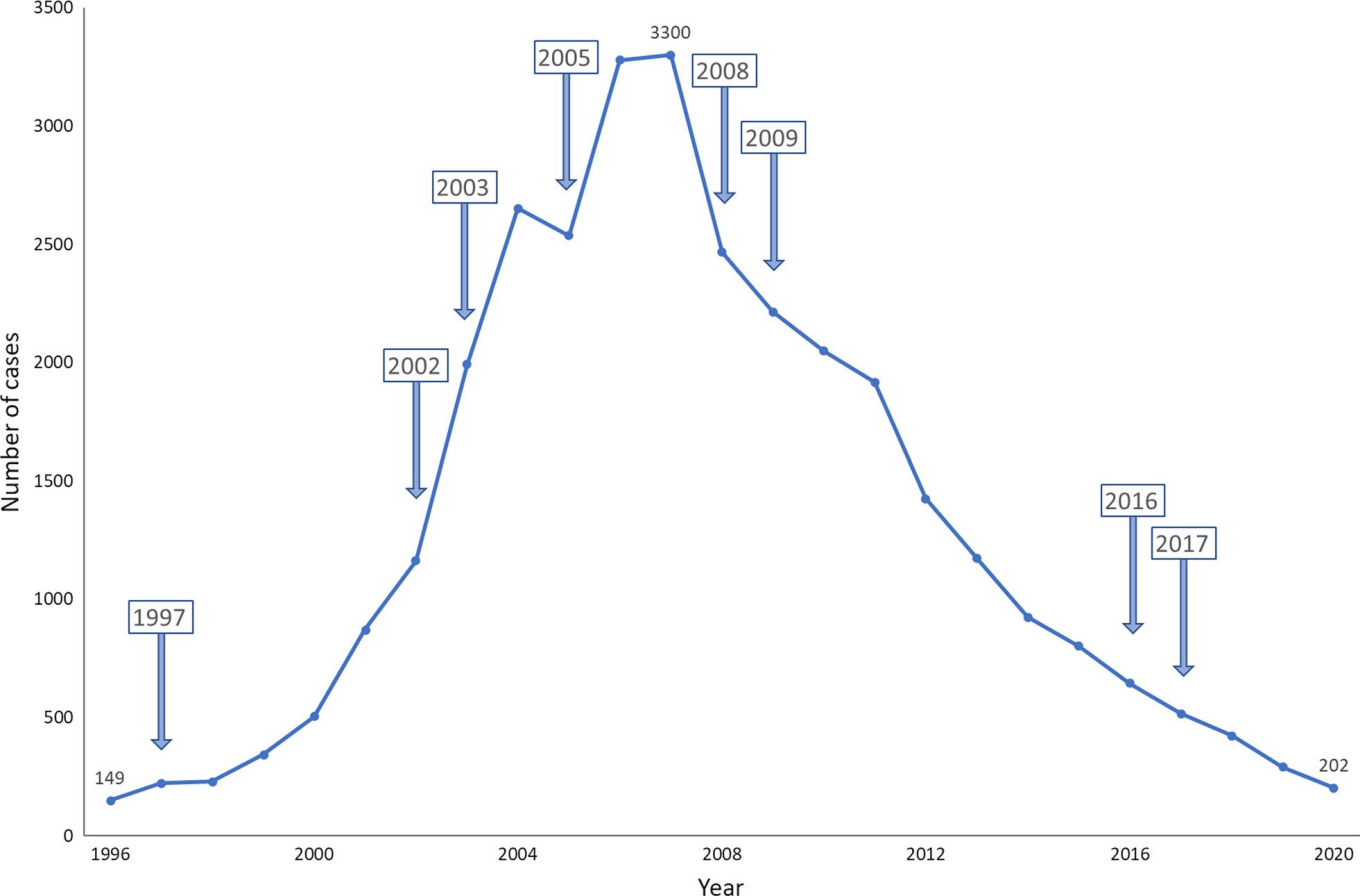
Reported cases of human rabies and the years of documents issued in China from 1996 to 2020. The years noted refers to the years in which the rabies-related documents were issued

In 2005, PR China established a National Rabies Surveillance System, establishing 15 sentinel counties in 6 provinces, making available robust data for the incidence of rabies in China from 2004. Not included Macau, Hong Kong, and Taiwan, 31 provincial-level administrative divisions (PLADs) in the Chinese mainland have reported human rabies cases (Table 1 and Figs. 2, 3).

**Table 1:** Distribution of reported human rabies cases in China

PLADs	2004	2005	2006	2007	2008	2009	2010	2011	2012	2013	2014	2015	2016	2017	2018	2019	2020
Nationwide	2651	2537	3279	3300	2466	2213	2048	1917	1425	1172	924	801	644	516	422	290	202
Hunan	523	379	443	334	229	200	152	162	118	83	65	75	64	71	78	55	59
Henan	130	136	159	196	116	108	89	99	86	81	78	78	82	52	42	37	27
Sichuan	16	49	191	372	172	102	82	74	68	67	45	36	20	27	21	19	20
Jiangsu	201	127	106	115	83	61	75	87	62	37	39	34	44	21	22	16	17
Guangxi	601	480	517	493	372	324	303	288	232	161	121	110	57	41	34	23	11
Hubei	224	184	218	151	90	120	84	87	55	38	41	37	32	39	34	22	11
Anhui	182	94	115	83	39	55	48	48	39	31	31	34	23	39	17	23	11
Guizhou	206	481	641	419	281	263	254	207	113	84	70	62	51	26	31	21	10
Shaanxi	1	3	1	0	0	26	21	42	45	49	31	25	14	17	18	13	7
Yunnan	13	11	38	65	108	73	134	118	84	79	73	57	48	32	21	16	6
Guangdong	245	306	387	334	319	330	301	202	158	140	85	48	44	23	18	8	6
Jiangxi	126	110	92	78	74	60	48	44	23	17	13	10	11	15	13	7	4
Zhejiang	74	61	58	57	38	31	25	18	14	8	9	8	19	14	13	5	4
Inner Mongolia	2	0	1	6	2	5	6	12	13	13	6	7	5	2	2	2	2
Shanxi	1	1	0	3	10	28	42	88	68	66	43	25	26	17	11	2	1
Hebei	7	12	86	144	110	135	121	91	66	68	56	44	31	8	11	4	1
Beijing	0	1	1	3	6	3	9	5	13	7	7	10	3	2	3	1	1
Gansu	0	0	0	0	0	1	0	0	0	9	4	11	8	9	1	1	1
Ningxia	0	0	0	0	0	0	0	2	3	8	14	8	2	1	1	1	1
Tianjin	0	3	4	18	10	11	4	8	9	4	5	2	5	2	0	1	1
Fujian	20	27	44	43	15	11	9	2	3	2	0	3	1	2	0	1	1
Chongqing	2	6	28	175	173	125	94	87	47	29	30	27	21	24	18	0	0
Shandong	64	60	133	139	111	92	74	91	67	55	32	37	25	23	8	0	0
Shanghai	3	1	7	7	1	3	3	7	4	1	3	0	1	2	5	0	0
Hainan	7	4	7	64	106	46	70	44	27	29	21	8	4	5	0	0	0
Tibet	0	0	0	0	0	0	0	0	0	0	0	1	1	1	0	0	0
Qinghai	0	0	0	0	0	0	0	0	1	1	0	0	1	1	0	0	0
Xinjiang	0	1	0	0	1	0	0	2	0	0	0	0	1	0	0	0	0
Liaoning	2	0	0	0	0	0	0	2	6	4	2	3	0	0	0	0	0
Jilin	1	0	0	1	0	0	0	0	0	0	0	1	0	0	0	0	0
Heilongjiang	0	0	2	0	0	0	0	0	1	1	0	0	0	0	0	0	0

PLADs: Provincial-level administrative divisions

Since 2007, when China reported its highest incidence (3300 reported human cases) of rabies in this century, rabies incidence has shown a downward trend year on year with the total number of rabies deaths nationwide falling to around 200 in 2020. The incidence of human rabies varies among PLADs in China with the current distribution of rabies being highly fragmented. While rabies incidence remains critical in some provinces including Hunan, Henan and Sichuan, other traditionally rabies affected PLADs of low-incidence, including Tibet, Qinghai, Xinjiang in western China and Liaoning, Jilin, Heilongjiang in north-eastern China have been no cases of human rabies in recent years. The incidence rate for rabies in traditional epidemic PLADs, including Guangxi, Guizhou, Guangdong, Chongqing, Shandong, and Hainan has declined considerably in recent years.

#### Rabies distribution in humans

The distribution of human rabies cases in PR China is geographically clustered [22] and from 1996 a rapid geographical expansion in counties reporting cases was observed. In 1996, 98 counties reported rabies cases (mainly from Guangxi, Guangdong, Hunan and Guizhou), this increased in 2003 with 190 counties from Jiangxi, Jiangsu, and Anhui reporting cases; and in 2007, 984 counties or 34% of the total number of counties in China reported cases. The number of counties reporting rabies cases subsequently decreased to 567 counties in 2014 and 143 counties in 2020 [23–25]. In ten years the area from which rabies cases occurred, grew from 162 counties in 2004 to 200 prefectures in 2014 [14]. These 200 counties are relatively densely populated and cover about one-fifth of China’s land area.

At least 27 of the 31 provinces in the Chinese mainland have foci for rabies infection [26] with the middle and lower reaches of the Yangtze River being most affected. Sustained efforts at control have resulted in the number of cases falling in China over the past decade. Since 2007, the number of human rabies cases in China has decreased by 94% by annual efforts, which is mainly due to the implementation of continuous surveillance and active prevention and control [27]. In 2020, the number of PLADs reporting human cases of rabies decreased to 21, of which 13 PLADs reported less than 10 cases.

Around 97.2% of rabies cases occur in rural areas of China [28]. Farmers, students, and children are the groups most afflicted by rabies, accounting for 63%, 17%, and 8% of the total cases respectively [29]. This is a different epidemiological profile from that observed in many middle- and low- income African and Asian countries, in which children account for 40% [6, 30]. Data for the period 2004‒2018, show that individuals aged 0–10 and from 40 to 60 account for a high proportion of people infected with rabies [31]. A 2007 survey among primary school students, showed the average rate of rabies exposure to be 9.2%, higher in boys than in girls, and higher in rural than in urban areas [32]. Over the past five years, the number of human rabies cases shows that infection has mainly affected farmers, with the proportion of students and children decreasing to 8% and 5% respectively [27]. The population at high risk in China has shifted to the age group of 40–70 years old, which accounted for 69% of cases in 2020. It is credible that education and improved awareness in children has resulted in this group being less exposed to rabies [33–35].

#### Human prophylaxis

Rabies is nearly 100% preventable. Many rabies victims were not able to obtain post-exposure prophylaxis in time. A study assessing 10,971 rabies cases in China presenting between 2006 and 2012 showed that only 35.0% of patients treated the wound, only 11.7% received post-exposure prophylaxis vaccination and only 3.0% were injected with rabies immunoglobulin [36]. In 2005, a retrospective survey of 885 rabies patients in 5 provinces of China showed that 60.6% of the patients failed to receive wound treatment, 49.0% of the patients did not have post-exposure vaccination and 96.16% did not receive immunoglobulin treatment [37].

Data for outpatient post-exposure prophylaxis at sentinel counties from the open access literature [37–44] were collected and are presented in Table 2. The number of outpatient visits for rabies post-exposure prophylaxis reported at surveillance sites has risen dramatically over the past two decades and is 15 times higher than it was initially. Vaccination rates are close to 100 percent, and antibody injection rates have increased significantly. These data confirm the increased accessibility and availability of rabies post-exposure disposal in China and the increased awareness of the population about rabies.

**Table 2:** Overall statistics for post-exposure outpatients at surveillance sites

	2005	2010	2012	2016	2017	2005–2011	2016–2018
Post-exposure population	92,045	233,700	269,201	1,281,340	1,509,225	1,377,941	5,978,300
Injury by dog (%)			82	79.5	76.3	83.9	75.2
Injury by cat (%)			16	13.0	14.7	11.4	17.5
Exposure category II (%)	24.0	52.6	50	48.6	48.3	48.5	49.9
Exposure category III (%)	57.0	40.6	43	44.8	46.0	39.0	44.2
Treatment by self (%)		17.0	21	26.6	32.2	16.3	28.4
Treatment in outpatient (%)		83.1	79	73.0	67.3	77.1	71.1
Wound treatment rate (%)	90.0						
Vaccination rate (%)	94.9	99.2	89	99.9	100.0	95.7	99.7
Whole course vaccination rate (%)		85.2	79	77.7	76.8	85.3	81.1
Antibody injection rate (%)	8.6	28.5	14.18	28.9	31.8		52.8
Injection rate for category III cases (%)						31.2	

However, post-exposure prophylaxis vaccination rates vary greatly in different regions of China. This may reflect regional differences in economic development, transportation infrastructure, per capita health resources, and knowledge of rabies. Analysis of 10,174 rabies patients from 2007 to 2017 showed that patients in the eastern region had a higher post-exposure prophylaxis vaccination rate (18.8%) than those in the western region (13.3%) [28]. The number of rabies clinics is an indicator of the accessibility of human post-exposure prophylaxis and the awareness of the community. Since 2016, the Chinese government has promoted the establishment of standardized rabies and in total, 29,106 rabies clinics had been set up by 2019 [45]. Despite the increase in the number of rabies clinics, regional differences still exist in China, more economically developed PLADs including Beijing and Shanghai are better served, than other less wealthy PLADs including Guizhou and Yunnan. With improved provincial development and services, the strengthening of public health messaging and education, and increased accessibility of rabies clinics, the number of outpatients acquiring rabies post-exposure prophylaxis has increased year on year.

### Dog rabies in China

#### Dog immunization and surveillance

To eliminate human rabies deaths, the most critical step is to achieve 70% immune coverage in dogs to interrupt transmission. If complete prevention is not carried out by immunization of dogs, the human health burden of rabies will persist in the long term. This is problematic in China, the high cost of dog vaccine, lack of awareness of dog vaccination, lack of professional veterinarians, and the large number of free-roaming dogs has resulted in the vaccination coverage rate of dogs that is lower than 70%.

Dog density and dog immunization rates at sentinel counties of China [37–44, 46] (see Table 3) shows that the density of dogs in China peaked in 2008 but has remained stable since 2016 at around 7 dogs per 100 people. The dog immunization rate is observed to markedly fluctuate at different surveillance sites but overall is around 30%. China CDC surveillance of canine rabies in typical epidemic areas shows the immunization rate of dogs in Guangxi Zhuang Autonomous Region in 1999 was only 3.9%; in Hunan, Henan, Guangxi, Guizhou, and Jiangsu 9.1% in 2004 and in Guizhou only 5.9% in 2005 [47]. Data from 2020, from national rabies surveillance sites, show the average immunization rate of dogs to only around 30% [27]. It is likely that differences in methods of calculation and given underestimates of the dog density in some provinces that even this low vaccination rate is overestimated [39].

**Table 3:** Overall statistics for dogs at surveillance sites

	2005	2005–2011	2006	2007	2008	2009	2010	2012	2016	2017	2016–2018
Dog density (dogs per 100 people)		7.0	14.1	10.2	21.8	16.3	12.3	10.9	7.0	7.2	7.0
Dog immunization rate (%)	42.0	36.4	6.0	9.6	20.4	18.0	32.9	2.9	37.6	30.9	

There are large differences in these estimates between rural and urban areas and between western and coastal PLADs. A study in 2005 suggested that in southern and central areas where the most cases were recorded, the average rate of dog vaccination was around 3% [11] and an exposure investigation in 2007–2016 in Chongqing reported only 4.0% of the domestic dogs related to the cases have been vaccinated [48]. In contrast in Fengtai District of Beijing, immunization coverage for 2006, 2007, 2008, and 2009 was 55.0%, 53.8%, 67.4%, and 54.4% respectively [49].

In 2009 a study compared virus-neutralizing antibody (VNA) in dog serums of different cities [50]. In metropolitan cities including Beijing and Shanghai, sera was shown to have a 100% positive rate of VNA; in economically developed including Shenzhen, Dongguan, Changchun a VNA-positive rate of 80% was observed; in the medium-sized city of Hengshui, the VNA-positive rate was 41.6% whole in six rural areas, the positive rate of VNA was extremely low, ranging from 1.2% to 2.8% [50]. A 2021 village survey in Guangxi, showed that only 19.1% of the households reported their dogs vaccinated against rabies [51].

The immunization coverage rate of rabies in China is showing an upward trend but disparities in coverage are observed in different regions, related to economic development; the immunization coverage rate of dogs in economically developed cities is higher than that in developed cities and rural areas. The risk of rabies resurgence in underdeveloped cities and rural areas in China is significant. Once the rabies virus spills over from natural foci, it can easily spread amongst the dog population and into humans. Rabies immunization of dogs in underdeveloped cities and rural areas needs to be addressed.

Rabies virus is widely distributed and present in dogs in China, and further in-depth rabies surveillance in dogs in China is necessary to obtain information on the prevalence of the disease in reservoir hosts for more efficient eradication efforts. A rabies surveillance plan implemented by the Ministry of Rural Agriculture that began in 2004 showed that of 185 animal brain tissues suspected of rabies submitted from 17 PLADs between 2004 and 2018, 144 were confirmed positive for the rabies virus. Dogs were the main animal found infected (68.8% of the total samples) [52]. In another study, 0.33% of the 10,118 samples collected during active surveillance of apparently healthy dogs in 7 PLADs in China were found to be positive for the rabies virus. Between 2003 and 2008, the rabies positive rate of brain specimens taken from dog meat restaurants across China was 2.1–3.3% [53]. In 2006–2008 in Guangxi, a survey showed 0.9–1.9% positivity for rabies virus in canine populations [54]. In 2006 in Hunan Province, the positivity rate was 2.8% [55]. From 2010, the National Reference Laboratory for Animal Rabies continuously collected and tested samples from rabies suspicious animals, of 212 samples submitted by 16 provinces and tested in the past decade, 80.2% were confirmed positive for rabies virus [56].

#### Dog management

Dogs are the main source of rabies in China, approximately 90% of human rabies exposure was caused by dogs [13, 36, 38, 47]. Dog keeping is very common in Chinese culture and as one of the six most representative livestock species, dog keeping is a centuries old practice. In China, there are no exact statistics for the number of dogs kept but estimates have placed the dog population at between 80 and 200 million dogs in China in 2005 [57]; 130 million in 2008 [50] and 75 million in 2009 [23]; the latter of which are likely to be underestimated. Most dogs are raised in rural areas and are used as working dogs, to guard homes or as companion animals, and in some PLADs are farmed or consumed as meat. Most dogs in rural areas are free-roaming and at great risk of acquiring and transmitting rabies. Free-roaming dogs are hard to capture and vaccinate [51]. In most rural areas of China, keeping dogs does not require registration, and the transportation of dogs is not restricted. In a survey in Shandong Province, only two of the 16 villages with rabies outbreaks were individual dogs immunized [23]. In cities, dogs are mainly kept as companion animals and these dogs are well served by veterinary services. Most cities have dog management legislation to enforce compulsory registration and vaccination. A dog owner will pay 50–80 Chinese Yuan (CNY) per dog for an annual canine rabies vaccine [36]. Rabies in cities is mainly transmitted by stray dogs [48]. In 2021, China issued the *(Revised) Law on Animal Epidemic Prevention*, which requires dog owners must ensure the animal is routinely vaccinated, and register the vaccination certificate (Table 4). This law provides a national legal guarantee for the elimination of dog-mediated human rabies in China. This is a very important leap in promoting dog management in China.

**Table 4:** Documents and policies on rabies control issued by China

Year	Source	Documents	Remarks
1951	GAC		A unified nationwide dog eradication led to a significant drop in the incidence of rabies [13]
1980	MoH	Notice on the control and elimination of rabies	The Notice proposed Regulations for the Management of Domestic Dogs
1984	MoH, MoALF, MoPS	Opinions on Strengthening the Prevention and Control of Rabies	The Opinions clarified the division of labor and functions of the departments, and put forward countermeasures for dog management, dog extermination, dog and immunization [10]
1989	SCNPC	Law on the Prevention and Control of Infectious Diseases	The administration of rabies prevention and control shall be handled by the departments of livestock, veterinary, health, and public security of governments at all levels under the provisions of the State Council
1991	MoH	Enforcement Measures for Law on the Prevention and Control of Infectious Diseases	The measures provided for the management of rabies control
1997	SCNPC	Law on Animal Epidemic Prevention	The law included rabies in the management of class II animal epidemics
2002	MoA	Rabies Prevention and Treatment Standard	Animal rabies was included in one of the seven standards of major animal diseases
2003	MoH, MoPS, MoA, SFDA	Notice on Strengthening the Prevention and Control of Rabies	The Notice required to carry out work in response to the rise of the rabies epidemic
2005	MoH	National Rabies Surveillance Program	The program carried out national routine surveillance and set up national surveillance sites
2005	MoH, MoA	Cooperative Mechanism for Zoonosis Diseases Prevention and Control	The mechanism aimed to strengthen the prevention and control of zoonotic diseases and strengthen coordination and cooperation between departments
2008	MoH	Diagnostic Criteria for Rabies	Rabies was included as one of the mandatory diagnostic criteria for 14 diseases
2009	MoH, MoPS, MoA, SFDA	Present Situation of Rabies Prevention and Control in China	The document summarized the work of rabies prevention and control in China
2009	MoH	Rabies Post-Exposure Treatment Standard	The Standard provided guidelines and requirements for human post-exposure prophylaxis
2012	the State Council	National Medium and Long-term Animal Epidemic Prevention Planning (2012–2020)	The Planning put forward assessment standards, requiring that rabies prevention and control should be strengthened in key areas, dog registration management should be improved, comprehensive immunization should be implemented, and sick dogs should be culled
2016	MoH	Technical Guidelines for Human Rabies Prevention and Control	The Guidelines provided scientific guidance on rabies prevention and control to grassroots agencies
2017	MoA	National Animal Rabies Prevention and Control Plan (2017–2020)	The plan calls for improving the diagnosis, surveillance, and immunization system, promoting the gradual elimination of animal rabies
2021	SCNPC	(Revised) Law on Animal Epidemic Prevention	The revised law requires dog owners must ensure the animal is routinely vaccinated, and register the vaccination certificate
2021	MoARA	National Animal Epidemic Surveillance and Epidemiological Investigation Plan	The Plan included rabies

GAC: Government Administration Council (now State Council); MoH: Ministry of Health (now National Health Commission); MALF: Ministry of Agriculture, Livestock and Fisheries (now Ministry of Agriculture and Rural Affairs); MoPS: Ministry of Public Security; SCNPC: Standing Committee of the National People's Congress; MoA: Ministry of Agriculture (now Ministry of Agriculture and Rural Affairs); SFDA: State’s Food and Drugs Administration (now National Medical Products Administration); MoARA: Ministry of Agriculture and Rural Affairs

### Strategies for rabies elimination in China

Since the founding of PR China has been proactive as regards elimination of infectious diseases, including rabies (Table 4). Under the guidance of various national policies and departments, the prevention and control of rabies has been actively promoted in many regions of China through the initiatives to regulate and manage pet treatment facilities and dog business activities, and to strengthen rabies immunization, surveillance and quarantine practices. The epidemic of rabies, as well as the transmission of rabies in humans, has been improved to a certain extent.

### Challenges to dog-mediated rabies elimination in China

While significant progress has been made in reducing the incidence of human rabies in China, universal access to post exposure treatment and vaccination of the dog population as an animal source control measures require strengthening. China yet faces numerous challenges to achieve the goal of eliminating dog-mediated human rabies by 2030 and if effective prevention and control measures are not taken, rabies will not be eliminated [58]. As in India and Indonesia, a massive canine rabies vaccination plan has not to date, been successfully and universally rolled out in China [59, 60]. While a large number of laboratory and basic epidemiological studies on rabies have been undertaken in China, information as to the efficacy of control and implementation of policy is lacking [61]. Implementation of large-scale dog vaccination in China faces many constraints including the determination of the large number of dogs to be treated, the lack of a unified dog registration system and a lack of human and material resources and a dependency on veterinary vaccination stations/provision in cities [62]. Besides, there is still a considerable gap in research on oral animal vaccine development and health economics evaluation of existing control strategies, which should be encouraged.

#### Inadequate animal surveillance

The number of domestic dogs kept in China has not been ascertained but there has been a year on year continuous increase in their number [63]. While many cities in China do have urban dog management regulations, policy implementation and compliance and lack of legal recourse mean that dog management depends on conscientious behavior from the owner. The efficacy of implementation varies from region to region and especially in rural areas. This is highly unfavorable for further prevention and elimination of rabies.

The diagnostic capacity of human and animal rabies laboratories needs to be strengthened. Underreporting is a major issue and animal rabies cases are greatly underestimated. Laboratory diagnoses of rabies in China are low with accounted for only 1.1% of cases in 2004–2012 [41]. The number of officially reported animal rabies cases between 2004 and 2018 was only 893, far lower than the 25,424 human cases reported in China during the same period [52]. In 2020, the number of animal rabies cases increased significantly, but only 64 of 1132 reported animal rabies were diagnosed in a laboratory. Animal rabies has been spreading in China, over the last decade new and recurring cases have been reported in areas where there had been no previous cases of rabies or only incidence, including, Heilongjiang, Xinjiang, Inner Mongolia, Tibet, Qinghai, and Taiwan [56, 64–66]. The temporary decrease in the number of human rabies deaths and the simultaneous increase in the spread of animal rabies is a typical example separation of human and animal health management [67]. Cross-department cooperation would overcome the gap of infrequent information sharing between the human and animal health sectors [56, 61] and applies to surveillance and reporting of all zoonotic infections in China including SARS, avian flu, leptospirosis, anthrax, and brucellosis [68]. A national system for centralized monitoring of animal diseases integrated with human health management would help to reduce the risk of animal to human transmission [69].

#### Rabies risk of wildlife and livestock

China has made significant efforts in the last two decades to restore the natural environment, enacting laws and regulations such as the *Wildlife Conservation Law* and *Regulation on Terrestrial Wildlife Protection*, and establishing more than 2000 nature reserves with habitats supporting wild animals that are an effective reservoir for rabies [38]. Surveillance has shown that over the past decade, wild animal rabies virus has spilt over into dog populations, and the risk of spillage is increasing and threatening public health in northern China. China is believed to be the principal source of rabies virus in Asia [70]; wild rabies viruses show extensive genetic diversity and are closely related to rabies virus strains circulating in surrounding countries and regions [15]. Although not common, rabid wild animal bites can cause human rabies cases; between 1996 and 2004, over 30 such human infections were reported derived from Chinese ferret badger bites from Zhejiang Province in eastern China [71, 72].

The Ministry of Agriculture and Rural Affairs has approved 10 domestic brands and 4 imported brands of inactivated canine vaccines albeit at inadequate yields for the vaccination of domestic dogs in China [73]. There is no commercially available rabies vaccine for livestock or wildlife available in China [74].

The risk of rabies in livestock caused by canines is of concern in China. In 2015 two animal rabies outbreaks, transmitted by dogs occurred in Shanxi Province, resulting in the death of 60 sheep, 10 cattle and 1 donkey [75]. Between February 2013 and March 2014, 7 domestic animal rabies outbreaks caused by wild carnivores occurred in Xinjiang Uygur Autonomous Region and Inner Mongolia Autonomous Region [76]. This highlights the need for wildlife surveillance and multisectoral collaboration in preventing spillover and transboundary rabies spread and justifies the need to promote immunization products and vaccines for both wild and domestic animals [77–79].

### Prospects for dog-mediated rabies elimination in China

Successful experiences of eliminating human rabies provide some promising opportunities for China. Reducing birth rates in dogs by sterilization and increasing dog immunization coverage rates offer the most effective methods for rabies control in China [58]. At the core of the strategy is to use a One Health approach to establish long-lasting herd immunity in dogs, by using mass vaccination campaigns for dogs, dog population management, and epidemiological surveillance, coupled with human post-exposure prophylaxis, and community education; these require interdisciplinary and intersectoral collaborations from human health sectors, animal health sectors, public security sectors, education sectors, commercial enterprise, local authorities and non-governmental organizations [80–84].

The infrastructure for the delivery of rabies control is not universally joined up across China, although one size is unlikely to fit all in a country as large and diverse as China. At present the public security sector is responsible for dog keeping in cities above the county level and the management of illegal dog keeping, the hunting, and killing of rabid and stray dogs; the animal husbandry and veterinary sector organizes the supply of veterinary rabies vaccine, is responsible for canine rabies vaccine injections, immunization record and issuance of “Dog Immunization Certificates” and surveillance of canine rabies epidemic; the health department is responsible for human rabies vaccination, patient rescue and treatment, and surveillance of human rabies epidemic and finally, the drug regulatory department is responsible for the supervision and management of the quality of human rabies vaccines.

This is changing, with demonstration projects showing different approaches. Rabies control in Guangzhou offers an excellent example of the application of One Health approach [85] (Box 1). In addition, grass-roots democratic politics with Chinese characteristics can have an impact and in 2019, Shanghai’s rural communities were enlisted in a rabies model village development project [86]. Similar policies could be promoted in 143 rabies-endemic counties across the country and offer a leap forward for the elimination of rabies in China.

However, the specific implementation of rabies prevention and control in different regions should be adjusted to local conditions. In the mountainous areas of central and Western China, attention should be paid to controlling the movement and breeding of stray dogs in the area; while in the plains areas, the strategy is to isolate villages where human rabies cases have been reported [87]. Methods to be implemented should divide the infected area and the buffer zone, to vaccinate all unimmunized dogs in the area, supplemented by the large-scale oral vaccine delivery to the wild animals in the area [88]. Oral vaccines are deemed safe and effective [89, 90]. Many countries in Europe use oral vaccine baits to significantly control wildlife rabies, especially fox rabies, and become rabies-free areas [91]. In the United States, in 1995, a national wildlife vaccination program was initiated by distributing oral rabies vaccines [92]; massive application of oral rabies vaccination eliminated rabies in many wild meso-carnivores, which contributed to the US being declared canine rabies free in 2007 [93, 94]. The United States (US) continues to prevent the spread of the raccoon rabies virus by maintaining oral rabies vaccine zones [92]. PR China and the US both have large areas of protected lands and ample recourses and the process of eliminating rabies in the United States provides a reference for China. The Chinese ferret badger plays an important role in China’s wildlife rabies reservoir [95–97] and rabies in this reservoir needs to be eliminated.

#### Access to human post-exposure prophylaxis

Socioeconomic status is a critical factor for access to human rabies post-exposure prophylaxis [98]. The economic cost may prevent rural residents from access to protection from rabies. In 2001, safer purified rabies vaccines became available in China but prices rose from 26 CNY/person to 156 CNY/ person [99]. In rural areas of the three high rabies prevalence provinces in China, the annual net income per capita in 2005 was 2496 CNY, while the total cost of complete post-exposure prophylaxis for a person was approximately 1150 CNY (full regimen vaccination 150 CNY, rabies immunoglobulin is around 1000 CNY) [99]. Between 2006 and 2012, the average annual income of rural residents in China was around 5490 CNY while the average total cost of complete post-exposure prophylaxis vaccination and rabies immunoglobulin vaccination was 300 CNY and 1000 CNY respectively [25]. As these expenditures are only partially subsidized by the national medical insurance system, the cost may be prohibitively expensive for many Chinese residents, especially farmers at high-risk of rabies [36].

The heavy economic burden is part of the reason why 67.2% of 244 patients with rabies in Guangdong province did not seek medical help [47]. In 2004, the per capita annual income in Guangdong Province was 3000 dollars, and the cost of rabies vaccine was 12.5 US dollars (USD) to 45.0 USD. In addition to the high cost of rabies post-exposure prophylaxis, limited access to appropriate rabies outpatient care and insufficient knowledge towards rabies prevention in the community also prevents patients in need from getting post-exposure prophylaxis [36, 100–102].

The rabies bite treatment rate in China has increased over the years, with the rate of outpatient treatment exceeding 70%, but standardization for outpatient treatment needs to be improved [44]. There are large differences in the accuracy of wound type categorization and the corresponding use of post-exposure prophylaxis in urban and rural areas [103]. Effective management in all parts of wound care and rabies vaccination decision making and ensuring nursing staff compliance and productivity, is essential [104].

#### Optimizing treatment for human rabies

Optimization of post-exposure schedules can save time and money by reducing clinic visits and vaccine doses and improving compliance of post-exposure prophylaxis. Schedules of post-exposure prophylaxis of rabies vaccine approved for marketing in China include a five-dose Essen regimen and a four-dose Zagreb regimen [24]. The immunogenicity of the Zagreb regimen is not inferior to the Essen regimen and has an acceptable safety profile [105, 106]. Excluding time and travel costs, individual medical costs can save 30% by simply changing the Essen regimen to the Zagreb regimen [107]. The IPC (Institut Pasteur du Cambodge, a 1-week, 2-site intradermal regimen on days 0, 3 and 7) regimen updated by WHO in 2018 requires 3 clinic visits and less vaccine dose and is even more economical than the Zagreb regimen [108, 109]. In the WHO position paper, the 2-week intramuscular regimen (1-1-1-1-0; 1-site intramuscular on days 0, 3, 7 and between day 14–28) and the three-week intramuscular regimen (2-0-1-0-1; 2-sites intramuscular on days 0 and 1-site intramuscular on days 7, 21) are also recommended as the simplified post-exposure prophylaxis. The IPC regimen shows the same safety and efficacy as the Essen regimen [110]. Compared with standard intramuscular injection, the IPC regimen can produce an equivalent immune response at a lower dose, to reduce the dosage and direct cost of the vaccine by 60–80% [111]. If the IPC intradermal regimen can fully replace the intramuscular regimen, China could save about 6 billion CNY in medical costs [24]. It has recently been shown that a short vaccination schedule of only 1 week is equally effective as the long vaccination schedule, which means that the post-exposure prophylaxis vaccination may complete within 1 week [112]. It is proposed to promote the use of the Zagreb regimen and explore the feasibility of the IPC regimen in China.

Studies have estimated that about 40 million people in China are injured by dogs and around 14 million people are vaccinated against rabies every year—a direct expenditure in vaccination estimated at 10 billion CNY [24, 39, 67]. In a case study of Dalian, a city where has been no human or animal rabies in the past two decades, half of the outpatients presenting did not require vaccination, and none of the remainder needed the five-dose full course vaccination [113]. Money saved could be redirected to ensure the dog vaccination target is met.

In the US an average of 55,000 people receive post-exposure prophylaxis treatment for potential rabies each year, and the per capita cost of rabies post-exposure prophylaxis is 3800 USD (four doses of rabies vaccine at 290 USD per dose and six to ten doses of rabies immunoglobulin at 312 USD per dose), which results in an estimated annual post-exposure prophylaxis cost of 209 million USD [114]. In the Philippines, the government provides rabies vaccines free of charge, but more than 85% of the patients who received the vaccination were not exposed to rabies [115].

A dynamic-decision tree model calculated the different rabies immunization strategies and corresponding cost-effectiveness in China, and concluded that 70% vaccination of dogs was the optimal strategy [116]. In the past 3 years, China has distributed about 37 million doses of inactivated rabies vaccine each year [74]. In a best-case scenario, the inactivated vaccines were sufficient to cover one-third of the estimated dog population in China, far from the 70% immunization coverage recommended by the WHO, and this does not account for the fact that some of the inactivated vaccines were deployed in cats. This estimated number is less than the 45 million dogs vaccinated in Latin America annually [33]. Meanwhile, in 2020, 78.55 million doses of human vaccine were deployed in China. China is the world’s largest human rabies vaccination market, and vaccinating humans does not interrupt transmission [61]. According to WHO, the costs needed to vaccinate one person (108.1 USD) could vaccinate 26 dogs (4.0 USD per dog) [117]. If the costs to deliver 14 million human vaccinations were used for massive dog vaccination, it would be sufficient to immunize 370 million dogs, equivalent to maintaining 70% coverage of dogs in China for four years. The Chinese government should take responsibility for protecting people’s lives, reducing their burden, preventing them from returning to poverty because of rabies and eliminating rabies in the most cost-effective way.

To reach the annual goal of 70% immunization coverage for dogs nationwide in China, it would cost only a quarter of current medical costs for people receiving post-exposure prophylaxis. The registration fee for keeping dogs levied by the city already includes the cost of vaccines. In rural areas, the government could pay for centralized procurement of veterinary vaccines so the overall burden on the government to implement mandatory vaccination for dogs is not large. China could solve the rabies problem once and for all at only a quarter of the cost of the human rabies vaccine.

## Conclusions

China has reached the final stage of eliminating rabies in dogs and humans, by launching multi-sectoral large-scale dog vaccination campaigns in endemic areas, coupled with dog population management, epidemiological surveillance, a more cost-effective human post-exposure prophylaxis, and community education, China is capable of eliminating dog-mediated human rabies by 2030.

Box 1. Two examples of dog-mediated rabies elimination in China **Multi-sectoral rabies control in Guangzhou**

The Guangzhou government actively uses the multi-sectoral model for rabies control, from 2 organizations involved in 1951 to 9 organizations involved in 2015, achieved remarkable results, reported zero cases in 2014 and 2015. In the twenty-first century, the Guangzhou government issued a dog management regulation and a code of practice for the prevention and treatment of rabies exposure. Guangzhou CDC is responsible for confirming the diagnosis and conducting epidemiologic investigations. Medical institutions and rabies clinics are responsible for identifying cases and providing post-exposure prophylaxis to those bitten. Rabies clinics are also used as surveillance points. The local CDCs, schools, and village committees are in charge of educating rabies-prone individuals The local police restrict the density of dogs through dog licensing. The Guangzhou agriculture and veterinary bureau immunize dogs regularly by injection. The Guangzhou industry and commerce bureau restricts trade in dogs and dog meat products. These 8 organizations and the Guangzhou CDC together, established the system of rabies surveillance and management. The in-depth and extensive inter-sectoral collaborations significantly reduced the number of rabies cases in Guangzhou. The approach is worth serving as a paradigm to guide other endemic areas.

**Model Village Development in Shanghai’s rural villages**

In 2019, Shanghai's rural communities have been enlisted in a rabies model village development project. They must follow a set of rules for managing the local canines that were developed by community veterinarians and enforced by the village's administrative officials. A door-to-door visit and registration of all home owned dogs, a government-led mass rabies vaccination day, and extra vaccination visits to houses that were not present during the mass vaccination were among the measures done. The local government provided full funding for rabies immunization in these settlements. A rabies vaccination certificate was issued to all immunized animals. Regular census surveys have been undertaken every quarter of the year for updated owned dog data since obligatory rabies vaccinations. After the application of these extraordinary policies, The model communities had 100 percent rabies vaccination coverage among their owned dog population and a lower number of stray dogs. Such a model village is an excellent pioneer experiment.

## Data Availability

Not applicable.

## Abbreviations

NTD: Neglected tropical disease
DALYs: Disability-adjusted life years
PR China: The People’s Republic of China
PLADs: Provincial-level administrative divisions
WHO: World Health Organization
HIV/AIDS: Human Immunodeficiency Virus/Acquired Immune Deficiency Syndrome
China CDC: Chinese Center for Disease Control and Prevention
CNKI: China National Knowledge Infrastructure
SARS: Severe Acute Respiratory Syndrome
CNY: Chinese Yuan
VNA: Virus-neutralizing antibody
US: United States
IPC regimen: Institut Pasteur du Cambodge regimen
USD: United States Dollar
